# Proteome dataset of mouse macrophage cell line infected with tick-borne encephalitis virus

**DOI:** 10.1016/j.dib.2019.105029

**Published:** 2019-12-19

**Authors:** A.L. Rusanov, A.A. Stepanov, V.G. Zgoda, A.L. Kaysheva, M. Selinger, H. Maskova, D. Loginov, J. Sterba, L. Grubhoffer, N.G. Luzgina

**Affiliations:** a V. N. Orehovich Institute of Biomedical Chemistry, Russian Federation; b Faculty of Science, University of South Bohemia, Branišovská 1760, 37005, České Budějovice, Czech Republic; c Institute of Parasitology, Biology Centre of the Czech Academy of Sciences, Branišovská 31, 37005, České Budějovice, Czech Republic

**Keywords:** Mouse macrophage cell line, TBEV, Proteomics, Protein, Mass spectrometry, Label-free quantification

## Abstract

We report the proteomic datasets on the mouse macrophage cell line PMJ2R infected with tick-borne encephalitis virus (TBEV) for two and six days. Data were acquired using shotgun ultra-high resolution mass spectrometry. Peptide identifications were done using the Mascot version 2.4 (Matrix Science), and quantification was performed by a label-free approach. Protein profiles of early (two days) and late (six days) stages of infection were compared between each other and the respective control samples.

Protein profiles of infected and control samples differed in the number of identified proteins and their relative abundances. Proteins detected in the TBEV-infected cells were involved in various processes related to the infection, including defense response against the virus, regulation of viral process, negative regulation of viral genome replication, RNA binding, or innate immune response. Also, proteins specific for the early and late stages of infection were identified.

**Table:** Specifications Table

Subject area	Biology
More specific subject area	Biochemistry, omics analysis, protein detection
Type of data	Spectra, figures
How data was acquired	Liquid chromatography-tandem mass spectrometric analysis was carried out using Q Exactive high-resolution mass spectrometer (Thermo Fisher Scientific, USA) coupled with an Ultimate 3000 Nano-flow HPLC system (Thermo Fisher Scientific, USA)
Data format	Raw, filtered, analyzed
Experimental factors	PMJ2R cells were infected with TBEV (strain Hypr) for 2 and 6 days
Experimental features	- Cultivation and infection of cells. - Cell lysis followed by protein precipitation. - Digestion of proteins. - LC-MS/MS analysis. - Data processing.
Data source location	Moscow, Russia
Data accessibility	Repository name: Proteomic data have been deposited to the ProteomeXchange Consortium via the PRIDE partner repository (http://www.proteomexchange.org/) Data identification number: PXD015164, PXD016227 Direct URL to data: https://www.ebi.ac.uk/pride/archive/
Related research article	Morphofunctional characteristics of the immune system in CBA and C57BL/6G mice. Shkurupiy V.A., Tkachev V.O., Potapova O.V., Luzgina N.G., Bugrimova J.S., Obedinskaya K.S., Zaiceva N.S., Chechushkov A.V. Bulletin of Experimental Biology and Medicine. 2011.150(6); 725–728.

**Table:** 

**Value of the Data** • This dataset, including raw data, can be used by other research groups interested in TBEV interactions with host cells, host defense mechanism to the infection, etc., or focused on the interaction of TBEV with immune cells. • Antigen-presenting cells (macrophages) play an important role in the initial phase of the development of tick-borne encephalitis, being the main reservoir of the virus during an incubation period and at the stage of early viremia. This determines the prospects of their use in the biomedical studies. • Comparative analysis of early and late stages of TBEV infection in PMJ2R cells provided a set of proteins for which their biological role should be further evaluated.

## 1 Data

The dataset contains “*.raw” and “*.mgf” files obtained through the high-throughput shotgun proteomics analysis of control mouse macrophage cell line PMJ2R (M2 and M6) after incubation for 2 and 6 days, and mouse macrophage cell line PMJ2R (M2 and M6) after infection with TBEV strain Hypr for 2 and 6 days (H2 and H6) (PXD016227 and PXD015164) (Table 1).

**Table 1:** Samples of mouse macrophage cell line PMJ2R.

Sample ID	Mouse macrophage cell line PMJ2R	Biological replicate	Technical replicate	Cultivation, days
H2_01_01	infected with TBEV Hypr	1	1	2
H2_01_02	infected with TBEV Hypr	1	2	2
H2_01_03	infected with TBEV Hypr	1	3	2
H2_02_01	infected with TBEV Hypr	2	1	2
H2_02_02	infected with TBEV Hypr	2	2	2
H2_02_03	infected with TBEV Hypr	2	3	2
H2_03_01	infected with TBEV Hypr	3	1	2
H2_03_02	infected with TBEV Hypr	3	2	2
H2_03_03	infected with TBEV Hypr	3	3	2
M2_07_01	control	7	1	2
M2_07_02	control	7	2	2
M2_07_03	control	7	3	2
M2_08_01	control	8	1	2
M2_08_02	control	8	2	2
M2_08_03	control	8	3	2
M2_09_01	control	9	1	2
M2_09_02	control	9	2	2
M2_09_03	control	9	3	2
H6_16_01	infected with TBEV Hypr	16	1	6
H6_16_02	infected with TBEV Hypr	16	2	6
H6_16_03	infected with TBEV Hypr	16	3	6
H6_17_01	infected with TBEV Hypr	17	1	6
H6_17_02	infected with TBEV Hypr	17	2	6
H6_17_03	infected with TBEV Hypr	17	3	6
H6_18_01	infected with TBEV Hypr	18	1	6
H6_18_02	infected with TBEV Hypr	18	2	6
H6_18_03	infected with TBEV Hypr	18	3	6
M6_10_01	control	10	1	6
M6_10_02	control	10	2	6
M6_10_03	control	10	3	6
M6_11_01	control	11	1	6
M6_11_02	control	11	2	6
M6_11_03	control	11	3	6
M6_11_04	control	11	4	6
M6_12_01	control	12	1	6
M6_12_02	control	12	2	6

All acquired raw files were converted to *.mgf files using Mass Hunter (version В 2.0), followed with database searching with Mascot version 2.4 (Matrix Science).

**Fig. 1:**
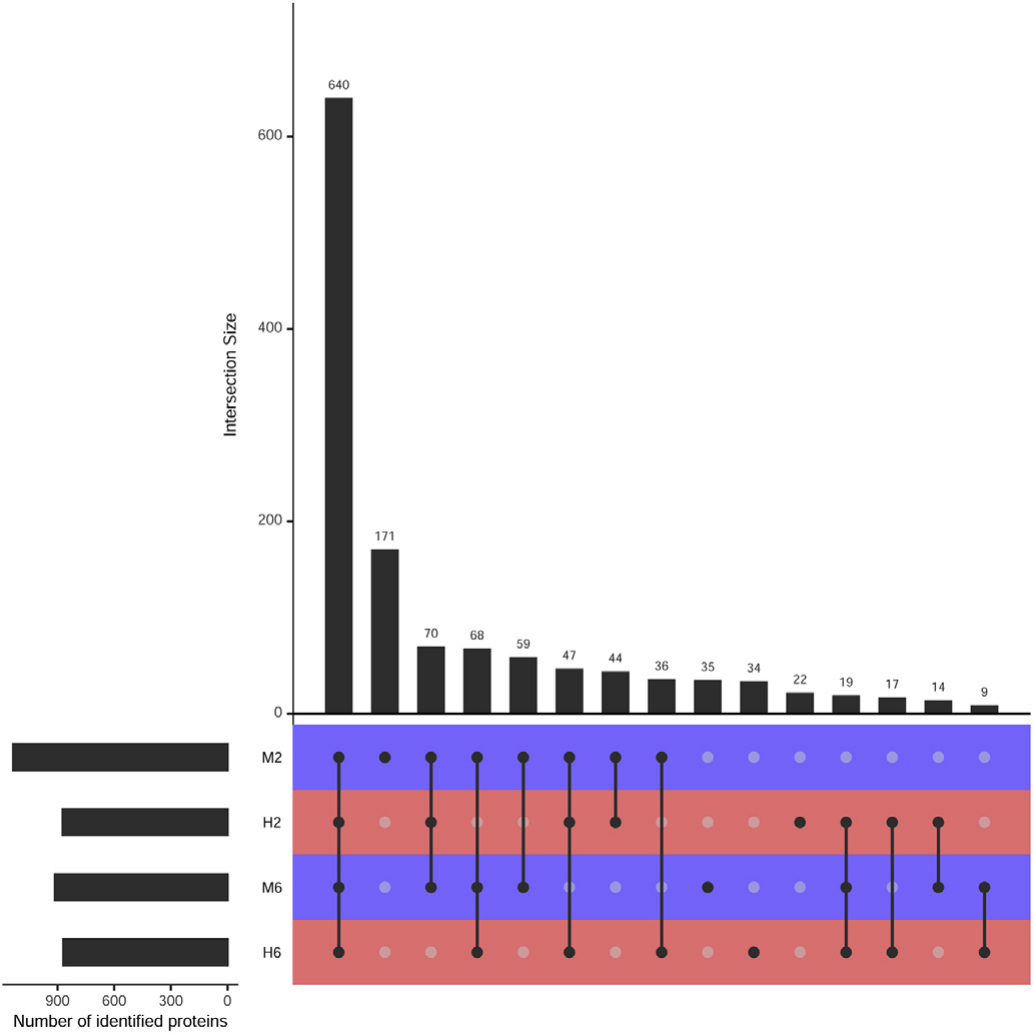
The UpSet diagram for all intersections of four samples, sorted by size. Dark circles in the matrix indicate sets that are part of the intersection. M2 and M6 – control samples at 2nd and 6th days, respectively. H2 and H6 – TBEV infected cells for 2nd and 6th days, respectively.

Infected and control cells were analyzed using a shotgun proteomic approach. Overall, 1212 proteins were identified in the control samples. The total number of protein hits in TBEV infected cells was 1020. Out of them, 640 proteins were common for all samples (Fig. 1). Synthesis of 265 host proteins was terminated in the course of TBEV infection (proteins were detected only in non-infected samples), and 73 newly synthesized proteins were discovered in infected cells (Fig. 1).

**Fig. 2:**
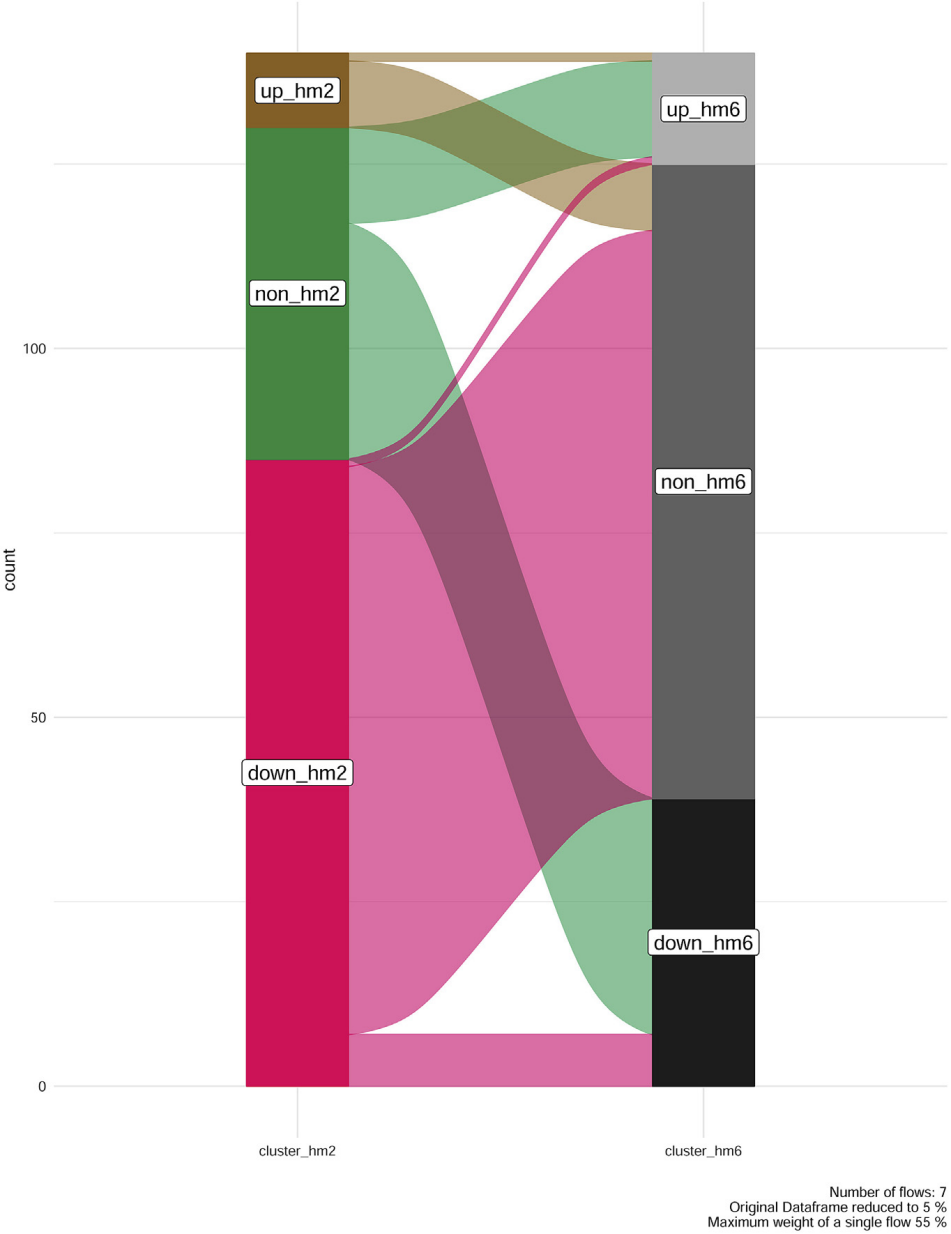
Alluvial diagram of changes in relative abundances of proteins in TBEV infected cells over time. “Up-hm2” – up-regulated proteins, 2 dpi; “Non-hm2” – constantly expressed proteins, 2 dpi; “Down-hm2” – down-regulated proteins, 2 dpi; “Up-hm6” – up-regulated proteins, 6 dpi; “Non-hm6” – constantly expressed proteins, 6 dpi; “Down-hm6” – down-regulated proteins, 6 dpi.

Changes in the abundances of proteins during infection are presented on Fig. 2.

## 2 Experimental design, materials and methods

### 2.1 Reagents

Acetonitrile, taurocholic acid sodium salt (TCA) and sodium chloride were from Merck (Germany). Formic acid was from ACROS Organics (USA). Modified trypsin was from Promega (USA). Tris-(2-carboxyethil)-phosphine (ТСЕР), methanol, trifluoroacetic acid (TFA) were from Fluka (Germany). Antibiotics-antimycotics, RPMI 1640 medium and fetal bovine serum were obtained from Biowest (France). L-alanyl-l-glutamine was from Biochrom (Germany). Dithiothreitol (DTT), iodoacetamide (IAA), β-mercaptoethanol, ammonium bicarbonate were from Sigma-Aldrich (USA).

### 2.2 Cell cultivation and cell infection

The murine macrophage cell line PMJ2R (ATCC CRL2458™) was cultivated in RPMI 1640 medium supplemented with 10% fetal bovine serum, 1% antibiotics-antimycotics (amphotericin B 0.25 μg/ml, penicillin G 100 units/ml, streptomycin 100 μg/ml), 1% L-alanyl-l-glutamine, and 50 nM β-mercaptoethanol. PMJ2R cells are derived from peritoneal macrophages immortalized by the infection with the J2 retrovirus. The donor was an adult *Mus musculus* female, strain C57BL/6J.

Cells were seeded on a 6-well plate at a density of 5 × 10^5^ (2 dpi) and 2.5 × 10^5^ (6 dpi) cells per well. Infection with low passage TBEV Hypr strain (GenBank U39292.1) was performed the next day at MOI 5.

### 2.3 Protein extraction

2 and 6 days post infection, cells were first washed three times with PBS and the lysis buffer (4% SDS in PBS, pH 7.4) was added afterwards. Cells were incubated with shaking on a vortex at room temperature for 20 min followed by incubation at 95 °C for 5 min. Then, samples were cooled down at ambient temperature and sonicated for 10 min. Proteins were precipitated using the methanol-chloroform method [1].

### 2.4 Sample preparation for MS analysis

Protein extracts (50 μg) were dissolved in 20 μL denaturation solution (5 M urea, 1% TCA, 15% acetonitrile, 100 mM phosphate buffer pH 6.3, 300 mM sodium chloride). Proteins were reduced by adding 5 μL of 25 mM TCEP in 0.1 M ammonium bicarbonate. After incubation at room temperature for 45 min, 5 μL of 300 mM IAA in 0.1 M ammonium bicarbonate was added. The reaction mixture was incubated in dark for 30 min. To quench the remaining IAA, 5 μL of 300 mM DTT in 0.1 M ammonium bicarbonate was added. The sample was diluted up to 200 μL with 0.1 M ammonium bicarbonate. Trypsin was added in enzyme:protein ratio 1:50. Digestion was performed at 37 °C overnight. After digestion, formic acid was added to the samples to the final concentration of 1%. Samples were centrifuged at 10 °C at 12.000×*g* for 10 minutes. The supernatant was purified using C18 StageTip [2].

### 2.5 LC-MS/MS analyses

Peptides were identified by HPLC-MS/MS using the high-resolution mass spectrometer Q Exactive (Thermo Fisher Scientific, USA) coupled to Ultimate 3000 Nano-flow HPLC system (Thermo Fisher Scientific). Peptides in the volume of 5 μL were applied on a trap column Acclaim PepMap C18 (Thermo Fisher Scientific) for 4 minutes in an isocratic flow of the mobile phase C (2% acetonitrile, 0.08% formic acid, 0.015% TFA) at a flow rate of 20 μL/min. Peptides were separated using the Acclaim PepMap C18 analytical column (75 mm × 150 μm, particle size 2 μm, pore size 100 Å) in the nano-flow mode in the linear gradient of the mobile phase A (0.08% formic acid, 0.015% TFA) and the mobile phase B (0.08% formic acid, 0.015% TFA in acetonitrile) at a flow rate of 400 nl/min at initial ratio А:В of 98:2. Separation was performed in the elution gradient from 2% to 35% of mobile phase B content for 80 minutes, followed by column washing at 90% of phase B for 10 minutes with subsequent system equilibration at initial gradient conditions for 20 minutes.

Detection of peptide signal was carried out in the dependent tandem scan mode with ionization source NSI (Thermo Fisher Scientific, USA). After prescanning of precursor ions with maximum accumulation time not more than 80 ms (or maximum accumulation value 3 × 10e6) with resolution R = 70K in the range of *m*/*z* 420–1250, 20 sequential tandem scans were made with maximum accumulation time not more than 120 ms (or maximum accumulation value 1 × 10e5) with resolution R = 17.5K with a fixed minimum range value (from *m*/*z* 220) and varying maximum range value depending on the resolved charge state. Ions with charge state z = 2^+^ … 5^+^ were selected for tandem scanning using the dynamic exclusion for the duration of one half-width of the chromatographic peak. Isolation of precursor ions was performed with the width of w = ±1 Th within the range from 9 to 17 s from the peak apex for the tandem scanning. Fragmentation was performed in the high-energy activation mode with rating 27% (per weight of *m*/*z* 400 and charge z = 2^+^) and variation per each scanning within ±15%. MS/MS spectra in a RAW format were processed in Mass Hunter version В 2.0 [3,4] Obtained data were deposited to the ProteomeXchange Consortium via the PRIDE [5] partner repository with the dataset identifiers PXD015164, PXD015164. Mass spectrometric measurements were performed using the equipment of “Human Proteome” Core Facility of the Institute of Biomedical Chemistry (Russia, Moscow).

### 2.6 Protein identification

Peak lists obtained from MS/MS spectra were identified using Mascot version 2.4 (Matrix Science). Protein identification was conducted against a concatenated target/decoy version of the Mouse Complement of the UniProtKB. The decoy sequences were created by reversing the target sequences in SearchGUI. The identification settings were as follows: Trypsin specific with a maximum of 2 missed cleavages; 20.0 ppm as MS1 and 0.05 Da as MS2 tolerances; fixed modification: carbamidomethylation (Cys); variable modifications: N-terminal proteins acetylation, and methionine oxidation (Met). Peptide Spectrum Matches (PSMs), peptides and proteins were validated at a 1.0% False Discovery Rate estimated using the decoy hit distribution.

### 2.7 Data analysis

Proteins for analysis were selected if number of identified peptides inside each biological replicate was greater than two. Qualitative diversity of proteins among samples was depicted using the UpSetR [6]. The Wilcoxon test was applied to compare the median of the emPAI values from three biological replicates of series H to the median of emPAI values from three biological replicates of series M independently for 2 and 6 day of cell culture [7]. The thresholds for significantly changed proteins were set to median p < 0.01 and median |log_2_FC| > 2. Thus, upregulated proteins had p < 0.01 and log_2_FC > 2, down-regulated proteins had p < 0.01 and log_2_FC < −2. Analyses were performed using an in-house script written in R (http://www.R-project.org).
